# All-Terrain Vehicle-Related Distal Upper-Extremity Injury Rates and Patterns

**DOI:** 10.1016/j.jhsg.2026.101044

**Published:** 2026-05-25

**Authors:** Janine Myint, Xinfei Miao, Subhas Gupta

**Affiliations:** ∗ Department of Plastic Surgery, Loma Linda University, Loma Linda, CA; † School of Medicine, California University of Science and Medicine, San Bernardino, CA

**Keywords:** All-terrain vehicles, Fractures, Hand surgery, Trauma, Upper extremity injury

## Abstract

**Purpose:**

All-terrain vehicle (ATV)-related upper-extremity injuries are commonly seen and treated in the emergency room. Despite existing research on patterns of injuries in ATV-related trauma, there has not been a comprehensive study on patterns of upper-extremity injuries from ATV use. The aim of this study was to examine the incidence, trends, and characteristics of ATV-related distal upper-extremity injuries across the United States.

**Methods:**

The National Electronic Injury Surveillance System database was utilized to investigate distal upper-extremity injuries specifically related to the use and operation of ATVs from 2013 to 2022. Demographic factors such as age, sex, race, and incident location were also examined. The injuries were further assessed to include diagnosis, involved body parts, and disposition. The injuries were also categorized based on their locations. Further subcategorization was done for forearm injuries to include ulna, radius, both, and unspecified.

**Results:**

Although there has been a decrease in emergency department visits for ATV-related injuries, there has been a gradual uptrend in the number of distal upper-extremity ATV injuries, with a peak of 19,973 in 2020. The number of male patients reported was approximately twice the number of female patients (68.87% vs 31.13%). Adolescents between the ages of 10 and 19 years experienced the highest incidence of ATV-associated distal extremity injuries (41.1% of reported). The sports field was the most common reported location of the incident (18.9%). Among all the injuries listed, fractures were the most common reported injury for each body part and overall. Of all reported forearm fractures, the radius was most involved.

**Conclusions:**

This study demonstrated a predominance of injuries in males and the 10–19-year age group and fractures as the most reported associated injury type. Given that the rate of ATV-related upper-extremity injuries has not followed the declining trend of overall ATV-related injuries, more attention should be focused on the effectiveness of upper-extremity injury prevention.

**Type of study/level of evidence:**

Prognostic III

All-terrain vehicles (ATVs) have gained widespread use in both agricultural and recreational settings since their introduction to the United States market in the 1970s.^1^, ^2^, ^3^ Despite a slight decline in fatal incidents associated with off-highway vehicles (OHVs) from 2016 to 2018, ATVs continue to pose significant risks to public health in terms of morbidity and mortality.^4^, ^5^, ^6^ The Consumer Protection Safety Commission (CPSC) reported 2,211 OHV deaths during a 3-year period from 2018 to 2021, with 1,591 associated with ATVs. Off-highway vehicle overturns and collisions are the leading causes of fatality, and common risk factors include young age, lack of helmet, male gender, use of alcohol, and inexperience.^5^^,^^7^ Approximately 30% of ATV-related injuries require hospital admission, and more than half of the patients with ATV-associated injuries are unhelmeted.^1^^,^^2^

Numerous studies have reported on ATV-related injuries to inform injury prevention strategies and improve patient outcomes. These injuries commonly include head trauma, chest injuries, extremity and facial fractures, and shoulder dislocations.^1^^,^^2^^,^^4^^,^^8^ Among the common injury sites, the upper extremity is frequently reported as the most commonly affected region.^2^^,^^4^^,^^8^ Given the prolonged rehabilitation associated with upper-extremity injuries, they often result in delayed return to work and incur significant medical costs.^9^^,^^10^

All-terrain vehicle-related upper-extremity injuries are commonly seen and treated in the emergency room, especially by plastic surgeons, orthopedic surgeons, and hand surgeons. However, despite existing research on patterns of injuries in ATV-related trauma, there has not been a comprehensive and up-to-date nationwide study on patterns of distal upper-extremity injuries from ATV use. The aim of this study was to examine the incidence, patterns, trends, and characteristics of ATV-related distal upper-extremity injuries across the United States using the US Consumer Product Safety Commission’s National Electronic Injury Surveillance System (NEISS) database in order to better understand these injury trends and outcomes nationwide and possibly contribute to the development of consumer safety recommendations.

## Materials and Methods

We collected patient records from the NEISS database, a national database managed by the United States CPSC, which gathers information on injuries related to consumer products. The database collects data from approximately 100 emergency departments (EDs) in the United States. Using this data, national estimates of injuries and demographics can be extrapolated.^2^^,^^8^^,^^11^ More information on the sampling procedure and the statistical basis for the calculation of national estimates based on the NEISS data can be found on the CPSC website.

In this study, the NEISS database was utilized to investigate distal upper-extremity injuries specifically related to the use and operation of ATVs during a 10-year period from 2013 to 2022. The queried injuries encompassed the lower arm (33), wrist (34), hand (82), and fingers (92). The query was conducted using NEISS product codes for different types of ATVs, including 3-wheeled ATVs (3,285), 4-wheeled ATVs (3,286), ATVs with more than four wheels (3,296), and ATVs with an unspecified number of wheels (3,287).

Exclusion criteria included mechanism of injury unrelated to ATV use and injury that arose from ATVs but was not directly associated with using it or caused by it. The accompanying narratives were reviewed by two reviewers to assess whether each case met the inclusion criteria. Additionally, due to the possible inaccuracies in the documentation process, the reviewers checked for inconsistencies in narratives and diagnoses and assigned multiple diagnoses if they were mentioned in the narratives.

Demographic factors such as age, sex, race, and incident location were examined in the analyzed dataset. The injuries were further assessed to include diagnosis, involved body parts, and disposition. Types of injuries that were examined included amputation, avulsion, thermal burns, contusion, crush injury, dislocation, fracture, hematoma, laceration, puncture, and strain/sprain. The injuries were also categorized based on their location, including the forearm, wrist, hand, and finger. Moreover, further subcategorization was done for forearm injuries to include ulna, radius, both, and unspecified.

This study was considered exempt from institutional review board approval due to the public nature of the NEISS database.

## Results

Between January 2013 and December 2022, there were an estimated 1,006,372 ED visits for ATV-related injuries, with 164,655 of those visits for distal extremity injuries (Table 1). Within the NEISS database, there were 3,550 reported ATV-associated distal upper-extremity injuries. However, approximately 258 were excluded from our analysis based on the exclusion criteria, resulting in 3,292 reported cases (Table 1). Since 2013, there has been an overall decrease in total ATV-related injuries nationwide (111,155 national injuries in 2013; 81,831 national injuries in 2022; relative difference 29,654). Overall, since 2013, there has been a gradual uptrend in the amount of ATV distal upper-extremity injuries, with a peak of 19,973 in 2020 (Figure).

**Table 1:** National ATV-Related Total Injuries and Distal Upper-Extremity Injuries From 2013 to 2022

Year	National estimate of total ATV-related injuries	National estimate of ATV-related distal upper-extremity injuries	Number of distal upper-extremity cases reported in NEISS
2013	111,155	15,898	306
2014	104,215	14,275	297
2015	108,357	15,992	276
2016	112,997	17,953	328
2017	104,803	15,834	307
2018	91,374	12,507	231
2019	90,957	18,380	338
2020	104,704	19,973	446
2021	95,979	17,706	431
2022	81,831	16,137	333
Total	1,006,372	164,655	3,293

ATV, all-terrain vehicle; NEISS, National Electronic Injury Surveillance System.

**Figure:**
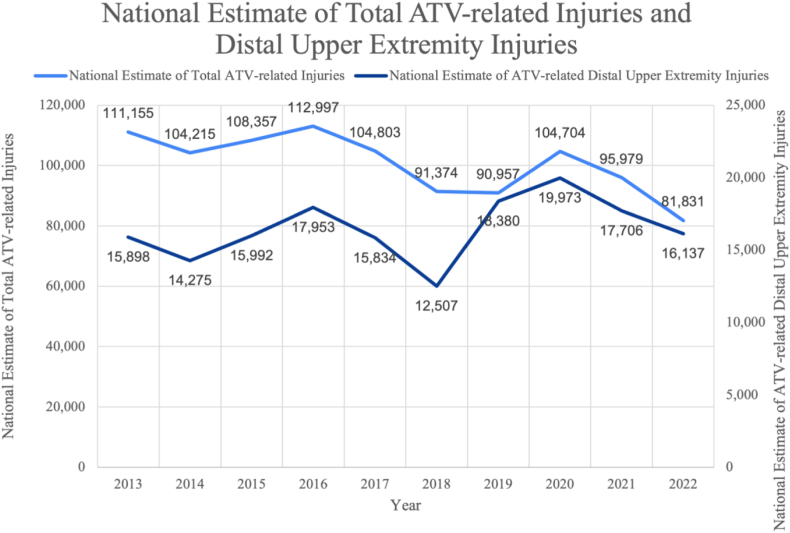
Trends in ATV-related injuries and distal upper-extremity injuries from the NEISS database from 2013 to 2022. This figure illustrates the patterns observed in ATV-related overall versus distal upper-extremity injuries based on data collected from the NEISS database between 2013 and 2022. The graph highlights the trends in these injuries, helping in understanding how the incidence of such injuries has evolved. ATV, all-terrain vehicle; NEISS, National Electronic Injury Surveillance System.

The number of male patients reported was approximately twice the number of female patients (68.87%, 2,268/3,292 vs 31.13%, 1,025/3,292) (Table 2). Adolescents between the ages of 10 and 19 experienced the highest incidence of ATV-associated distal extremity injuries (41.1% of reported, 37.3% of national estimate), followed by patients between the ages of 20 and 29 (19% of reported, 19.5% of national estimate) (Table 2). Of the 3,293 cases, 1,612 (49%) cases did not have a specified location where the specified injury took place. The most common reported location of incidents was sports fields (18.9%, 625), followed by home (557, 16.9%). Of the reported cases, most were examined, treated, and discharged (2,833, 86%) and another 10% (327) were admitted.

**Table 2:** Patient Demographics and Disposition–Reported and Estimated National Totals

Characteristics	Reported injuries (n%)	Estimated national total injuries (weighted %)
Age (y)		
0–9	412 (12.5%)	18,162 (11.1%)
10–19	1,355 (41.1%)	61,158 (37.3%)
20–29	626 (19%)	31,926 (19.5%)
30–39	455 (13.8%)	24,905 (15.2%)
40–49	218 (6.6%)	13,155 (8%)
50–59	124 (3.8%)	7,379 (4.5%)
>60	103 (3.1%)	7,432 (4.5%)
Sex		
Male	2,268 (68.87%)	115,657 (70.24%)
Female	1,025 (31.13%)	48,998 (29.76%)
Race		
African American	286	
American Indian	11	
Asian	7	
Native Hawaiian/Pacific Islander	3	
Not specified	874	
Other	153	
White	1,959	
Location of incident		
Farm	16	
Home	557	
Public	269	
School	2	
Sports fields	621	
Street	216	
Unknown	1,612	
Disposition		
Held for observation	26	
Left without being seen	29	1,323
Treated and admitted/hospitalized	327	10,413
Treated and transferred	78	4,855
Treated/examined and released	2,833	146,961

There were 1,407 forearm injuries (40%), 907 wrist injuries (25.7%), 733 hand injuries (20.8%), and 479 finger injuries (13.6%) (Table 3). Among all the injuries listed, fractures were the most common reported injury for each body part and overall, occurring in 49.1% of all distal upper-extremity injuries (1,740 out of 3,541) (Table 4). Contusions/abrasions were the second most common injury, occurring in 18.1% (641/3,541) of reported injuries (Table 4). Other common injuries included “other,” which was usually diagnosed as pain to the reported body part, followed by strain/sprain, and laceration. Of the 883 reported forearm fractures, 40.5% involved the radius, 7.6% involved the ulna, 17% involved both bones, and 34.9% were unspecified.

**Table 3:** Injured Body Part and Diagnosis, Reported and Estimated National Totals

Body part and diagnosis	Reported cases	National estimate
Lower arm	1,407	52,830
Amputation	3 (0.21%)	
Avulsion	6 (0.43%)	
Burns, thermal	4 (0.28%)	
Contusions/abrasions	296 (21.04%)	13,232
Crushing	11 (0.78%)	
Dislocation	6 (0.43%)	
Foreign body	3 (0.21%)	
Fracture	883 (62.76%)	27,763
Laceration	98 (6.97%)	5,288
Nerve damage	3 (0.21%)	
Other	69 (4.9%)	
Puncture	4 (0.28%)	
Strain/sprain	14 (1%)	
Wrist	907	54,409
Amputation	4 (0.44%)	
Avulsion	1 (0.11%)	
Contusions/abrasions	78 (8.60%)	3,858
Dislocation	8 (0.88%)	
Foreign body	1 (0.11%)	
Fracture	425 (46.86%)	30,937
Laceration	12 (1.32%)	
Other	109 (12.02%)	5,417
Strain/sprain	271 (29.88%)	13,956
Hand	733	36,785
Amputation	3 (0.41%)	
Avulsion	6 (0.82%)	
Thermal burns	13 (1.77%)	1,996
Contusions/abrasions	238 (32.47%)	12,600
Crushing	20 (2.73%)	
Dislocation	6 (0.82%)	
Foreign body	3 (0.41%)	
Fracture	227 (30.97%)	9,603
Hematoma	5 (0.68%)	
Laceration	111 (15.14%)	6,471
Other	60 (8.19%)	2,889
Puncture	2 (0.27%)	
Strain/sprain	32 (4.37%)	1,660
Finger	479	25,347
Amputation	42 (8.77%)	2,307
Avulsion	13 (2.71%)	
Thermal burns	2 (0.42%)	
Contusions/abrasions	29 (6.05%)	1,575
Crushing	11 (2.30%)	
Dislocation	14 (2.92%)	
Foreign body	4 (0.84%)	
Fracture	205 (42.80%)	9,811
Hematoma	3 (0.63%)	
Laceration	98 (20.46%)	6,655
Other	60 (12.53%)	1,230
Strain/sprain	35 (7.31%)	2,279
Puncture	1 (0.21%)

**Table 4:** Commonly Reported Diagnoses

Diagnosis	Reported cases (%)
Amputation	52 (1.5%)
Avulsion	26 (0.7%)
Burns, thermal	19 (0.5%)
Contusions/abrasions	641 (18.1%)
Crushing	42 (1.2%)
Dislocation	31 (0.9%)
Foreign body	11 (0.3%)
Fracture	1,740 (49.1%)
Laceration	319 (9.0%)
Nerve damage	3 (0.08%)
Other	298 (8.4%)
Puncture	7 (0.2%)
Strain/sprain	352 (9.9%)
Total	3,541

## Discussion

All-terrain vehicles have become an increasingly common recreational activity for families and adolescents and have even become household vehicles. There have been several studies on ATV-related injuries, but none on the nationwide incidence of distal upper-extremity injuries. This study addressed gaps in ATV injury literature, and the findings of this study contribute to the increasing concern regarding injuries associated with ATV use, which have seen an alarming increase since the introduction of ATVs to the United States in the 1970s. Despite this concerning trend, the implementation of safety guidelines and regulations of ATV operation has fallen behind and mainly been self-regulated.^9^^,^^12^

Based on the annual national estimate of ATV-related injuries, there has been a statistically significant decrease in the number of ED visits for overall ATV-related injuries (*R* = 0.76, *P* = .019). However, our study revealed an overall increasing trend, although not statistically significant (*R* = 0.38, *P* = .2823), of distal upper-extremity injuries requiring ED visits between 2013 and 2022, with its peak between 2020 and 2022. Despite the overall declining trend in ATV-related injuries, the incidence of distal upper-extremity injuries has not followed the same pattern, suggesting that there may be a need for increased awareness and focus on upper-extremity safety.

In this study, ATV-associated distal upper-extremity injuries were found to occur predominantly in male individuals and in the 10–19-year-old age group. These findings agree with many prior studies that investigated various ATV-related injuries.^8^^,^^9^^,^^13^, ^14^, ^15^ Part of this gender disparity is due to a higher number of males participating in the use of ATVs.^8^ Numerous studies have also documented the male propensity for risk-taking behaviors with resulting traumatic injuries, explaining the two to one proportion of male to female injuries.^7^^,^^16^ The higher rate of upper-extremity injuries in the 10–19-year age group is also alarming, especially because the average age of OHVs has been reported to be 34.9 years old.^8^^,^^17^ The increased rate of injuries in this specific age group can likely be attributed to lower physical and mental maturity, resulting in a higher propensity for risk-taking behaviors without knowledge of safe driving and operation of vehicles. This gender and age propensity in ATV-related upper-extremity injuries underscores the importance of targeted educational and preventive interventions that specifically address male riders, younger riders, and the risk behaviors they exhibit, aiming to promote safer ATV operation practices.

Multiple studies on ATV-related injuries have found that fractures are often the most common reported injury.^2^^,^^8^ In this study, among all distal upper-extremity injuries identified, fractures were the most common type, consisting of approximately 49.1% of all distal upper-extremity injuries (Table 4). Similar to what has been reported in other studies, this study found that the majority of reported fractures occurred in the lower arm (883, 50.7%) and the wrist (425, 24.4%). Traumatic distal upper-extremity injuries have been demonstrated to be associated with significant immobility, delayed return to work, loss of workforce, prolonged recovery period, and increasing direct and indirect costs on both an individual and national level.^8^^,^^18^ These injuries result in significant morbidity, prolonged recovery, and economic burden.^19^

Injury prevention in the current literature was mainly focused on helmet use, which has been proven to reduce traumatic brain injuries, neck injuries, and mortality in ATV-related trauma.^20^^,^^21^ Given that the rate of ATV-related upper-extremity injuries has not followed the declining trend of overall ATV-related injuries, greater attention should be directed to the effectiveness of upper-extremity injury prevention such as rider education and mandating the utilization of safety equipment in the form of arm restraints that keep the upper extremities in the vehicle, especially during rollovers. In addition to reinforcing helmet use mandates, other policy changes, such as implementing age and licensing restrictions and prohibiting children from operating adult-size ATVs, should also be prioritized.

Several limitations were encountered in this study. First, the NEISS database is a public database that may contain incorrect or incomplete reports and narratives. Utilization of this database in a retrospective, observational fashion may result in underreporting or overreporting of injury because methods of reporting are not standardized. For example, distal radius and ulna fractures have been noted to be classified within both wrist and lower arm injuries. The authors tried to adjust for this by reclassifying them as lower arm injuries when noted in the narratives. Additionally, this study is susceptible to selection bias because the NEISS database is limited to injuries that were seen in the EDs. Other medical settings such as urgent cares, primary care providers, or outpatient clinics were not included. This could result in an underestimation of the ATV-associated distal upper-extremity injuries and/or overestimation of the severity of injuries from 2013 to 2022. Additionally, the NEISS database obtains injury reports from a selected 100 EDs in the United States. Therefore, the reported injuries and data may not be entirely accurate for nationwide injuries.

This study provides valuable insights into the rates and patterns of ATV-related distal upper-extremity injuries in the United States. Key findings include predominance of injuries in males and the 10–19 age group, and fractures as the most reported associated distal upper-extremity injury type. Despite a decline in overall ATV-related injuries, distal upper-extremity injuries have not followed the same downward trend, suggesting a need for increased attention and prevention strategies in this group of injuries in order to mitigate the significant impact on individuals and public health.

## Conflicts of Interest

No benefits in any form have been received or will be received related directly to this article.
